# Study on Influencing Factors of High-Temperature Basic Characteristics of Iron Ore Powder and Optimization of Ore Blending

**DOI:** 10.3390/ma15093329

**Published:** 2022-05-06

**Authors:** Shuangping Yang, Haijin Liu, Haixing Sun, Tiantian Zhang, Shouman Liu

**Affiliations:** School of Metallurgical Engineering, Xi’an University of Architecture and Technology, Xi’an 710055, China; yang_sping@163.com (S.Y.); 18434162589@163.com (H.S.); ztt15129236642@163.com (T.Z.); liusm617100@163.com (S.L.)

**Keywords:** low-silicon ore, optimization of ore proportioning, assimilation liquid, phase fluidity

## Abstract

In order to explore the reasonable ore blending of low-silicon magnetite in sintering, it I necessary to realize the efficient utilization of low-silicon ore, further reduce cost, and increase yield. In this study, based on the high-temperature basic characteristics of iron ore powder used in the experiment, sinter pot tests were carried out with different low-silicon ore ratios, and the microstructure of the sinter was observed by scanning electron microscopy (SEM) and energy spectrum analysis (EDS) to determine the optimal matching law of low-silicon ore. The result showed that SiO_2_, Al_2_O_3_, and burning loss in iron ore powder composition were positively correlated with its assimilation, whereas MgO and basicity R_2_ were negatively correlated with the assimilation of iron ore powder. When the ratio of low-silicon ore was not more than 35%, increasing the ratio of hematite improved the liquid production and increased the production of acicular calcium ferrite. Therefore, the optimization of ore blending based on assimilation can improve the quality of sinter and strengthen the sintering process. This study has certain reference significance for the industrial production of low-silica sintering.

## 1. Introduction

In view of the characteristics of wide, miscellaneous, and poor iron ore in China [1,2,3,4], most available ore is extremely poor, and the types of ores are complex, with low iron grade few available iron ore resources. At present, the problem is to ensure the production and quality of sinter under the premise of reasonable blending of high-quality ore and inferior ore to improve the metallurgical properties of sinter and sintering production, reduce production costs, and achieve efficient utilization of inferior ore. In recent years, the performance of iron ore at normal temperature have been studied for ore blending [5]. Due to the change of ore types, the composition and proportion of ore blending change greatly, and it is difficult to refine and adjust the ore-blending scheme through a large number of sintering experiments. With the increase in research scope and depth, Shengli Wu et al. found that the physicochemical properties of iron ore powder at high temperature were closely related to the sintering process [6,7]. Kasai and Saito studied the melting behavior of iron ore during sintering and found that controlling the particle size of iron ore and the range of Al_2_O_3_ can reduce the assimilation temperature [8]. Haofang Wu, Yanzhong Jia, and Delan Liang studied the effect of physical and chemical properties of iron ore powder on assimilation [9]. It was found that the assimilation temperature showed a linear positive correlation with the SiO_2_/Al_2_O_3_ content ratio and LOI value, as well as a linear negative correlation with T_Fe_ content. Lijuan Yan, Shengli Wu, and Juan Zhu explored the influence of mineral morphology in iron ore powder on assimilation temperature [10,11] and found that the deterioration of the chemical composition of iron ore powder had significant influence on assimilation ability. When studying the assimilation ability of iron ore powder, Guoping Luo and Guolong Sun found that crystal water could increase the porosity and improve the kinetic conditions of the reaction in the dehydration process [12]. By exploring the high-temperature and sintering melting characteristics of iron ore powder, Shengli Wu, Dauter, and Miaolian Bian optimized ore blending to achieve suitable high-temperature characteristics. According to a quantitative ore-blending design scheme, a sinter pot test was carried out to obtain excellent sintering indices [13,14]. Guocheng Zhang and Guoping Luo verified the optimal configuration of limonite in the sintering process according to the microstructure of sinter by simulating the content of adhesion powder and theoretical liquid production under different limonite ratios, studied the influence of Brazilian high-silicon mixed powder on sinter quality, and proposed a reasonable ore-blending scheme combined with the basic characteristics of sintering [15,16].

In this study, the assimilation of iron ore powder and its influencing factors were explored, and an ore-blending scheme was formulated according to the assimilation of iron ore powder. Based on the high-temperature basic characteristics of iron ore powder, sinter pot tests were carried out with different low-silicon ore ratios. The rationality of ore blending was verified by analyzing the microstructure of sinter, which provided experimental guidance for the reasonable ore blending of low-silicon sinter raw materials.

## 2. Materials and Methods

### 2.1. Materials

A total of 10 kinds of iron ore powder samples were used in this study. Samples A1~A6 were domestic fine iron powder, B1~B2 were foreign iron ore powder, and C1~C2 was sinter return fines. The chemical composition of iron ore powder, coke powder composition, and flux composition are presented in Table 1, Table 2 and Table 3, respectively.

The chemical composition of iron ore powder samples presented in Table 1 shows that the iron grade of A1 iron concentrate is 62.62%, the SiO_2_ and MgO contents of the sample are 2.25% and 1.66%, and the binary basicity is less than 0.5, which is characteristic of acidic ore powder. Sulfur, phosphorus, and other impurities are within the normal range, and the LOI is 0.83%, which is characteristic of high-quality low-silicon magnetite. In this study, the low-silicon iron powder A1 distributed in Qinghai Province of China was used as the main ore for ore blending research. Because the silicon content of the ore is low and unstable, the average particle size is less than 0.075 mm, the liquid-phase production in the sintering process is low, and the assimilation temperature is high, which can easily lead to unstable sinter; therefore, it is difficult to ensure the smooth operation of the blast furnace.

It can be seen from Table 2 that the average particle size of low-silicon ore A1 is 31 μm, the average particle size of returned ore C1 and C2 is more than 3 mm, the average particle size of specularite A3 and A5 is less than 75 μm, and the average particle size of other iron ore powders is 2~5 mm. The particle size composition of iron ore powder has a considerable influence on the granulation performance of sintering raw material, the permeability of the sintering process, and the quality of the sinter. Ore powder with a 2~5 mm particle size should be added in as high a proportion as possible in the sinter so as to make the material ball more uniform, increase the intensity of the sinter, and improve the yield and quality. The iron ore particles of 0~1mm are difficult to granulate and mainly play a role in adhesion. The higher the ratio of mixing, the lower the sintering productivity.

Due to its high carbon content; moderate ash and volatile substance content; and low content of harmful elements, such as P and S, anthracite was used as the most suitable carbon material in the sintering process. Chemical analysis results of the coke used are presented in Table 3.

It can be seen from the flux composition in Table 4 that the CaO content is 89.58%, the SiO_2_ and MgO contents are 0.57% and 0.17%, respectively, and the LOI value is 9.68%.

### 2.2. Methods

#### 2.2.1. Assimilation

**Figure 1:**
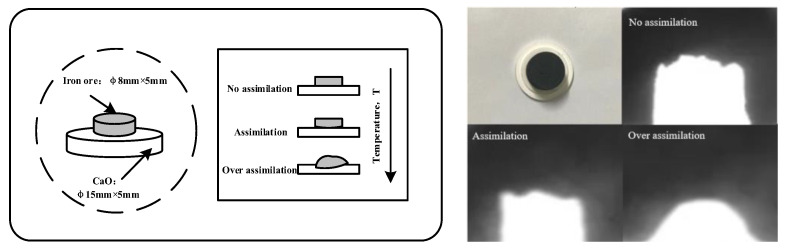
Schematic diagram of assimilation.

Schematic diagram of assimilation is shown in Figure 1 and the specific experimental process is as follows: The iron ore powder sample was ground to a particle size of less than 75 µm (−200 mesh) and placed in a drying chamber for full drying. Amounts of 0.8 g iron ore powder sample and 2.0 g CaO (analytical pure) reagent were used to prepare an 8 mm × 10 mm ore cylindrical sample and a 25 mm × 4 mm CaO gasket sample on a tableting machine. The iron ore powder sample was placed on the CaO gasket in an infrared horizontal high-temperature furnace to ensure that the bottom surface of the iron ore powder was fully in contact with the CaO gasket plane. When the horizontal tube furnace was heated in an oxidizing atmosphere, the heating rate of the furnace was 7 °C/min, and the sample was calcined in an oxidizing atmosphere. The experiment was finished when the corrosion phenomenon occurred on the contact surface of the iron ore powder and CaO, and the minimum temperature of this morphological change of iron ore powder was determined.

#### 2.2.2. Liquid-Phase Fluidity

**Figure 2:**
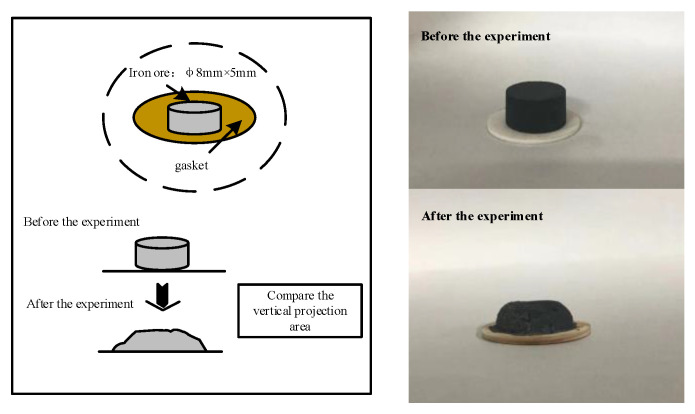
Schematic diagram of liquid-phase fluidity.

Schematic diagram of liquid-phase fluidity is shown in Figure 2 and the specific experimental process is as follows: The particle size of the iron ore powder sample was less than 75 µm (−200 mesh). The sample was dried, and CaO (analytical) reagent was added to adjust the basicity to R = 3.0 and mixed with a mortar and pestle. Next, 0.8 g of the mixed material was taken for further analysis, and a hydraulic machine was used to pressurize the mixed material to prepare φ8 mm × 10 mm cake samples. The cake samples were calcinated on the gasket in the infrared horizontal high-temperature furnace and held at 1280 °C for 5 min. An oxidizing atmosphere was maintained throughout the experiment. The small cake sample was calcinated in the horizontal high-temperature furnace and removed slowly after cooling. The liquid-phase fluidity index of iron the ore powder sample was calculated using Equation (1):

FLP = Aa − Ab/Ab(1)

where Ab and Aa are the vertical projection area of the sample before and after the experiment, respectively (mm^2^). If the cake area does not change before and after calcinating, the fluidity index is 0.

## 3. Results and Discussion

### 3.1. Assimilation Tests

It can be seen from Table 5 that the lowest assimilation temperature of each mineral powder is uneven. By comparing the assimilation temperature, it was found that the lowest assimilation temperature of mineral powder A2 was 1252 °C, and that of C1 and A5 exceeded 1300 °C. The assimilation ability of this mineral powder was the worst, and the order of assimilation ability, from strong to weak, was A2 > B2 > A6 > A4 = A1 > B1 > 3 > C2 > A5 > C1.

### 3.2. Liquid-Phase Flow Tests

**Figure 3:**
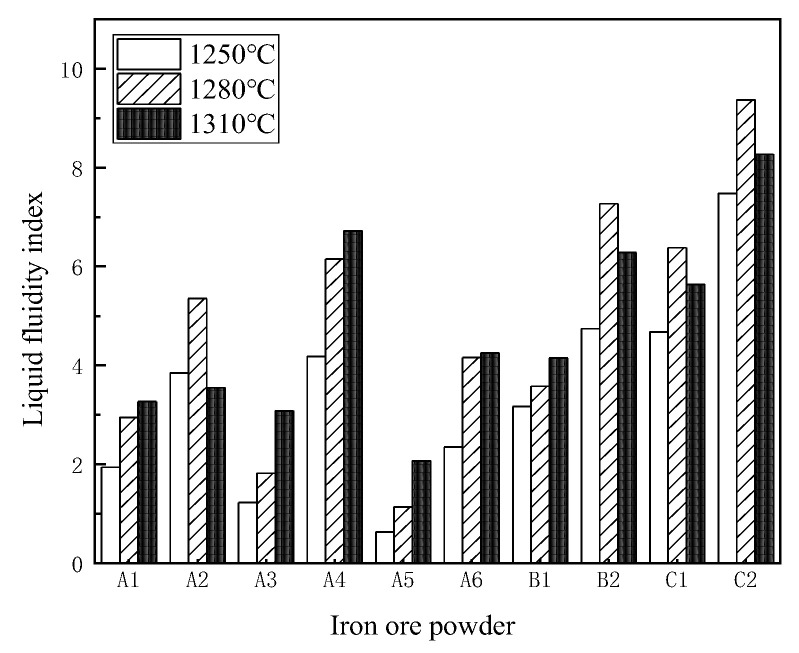
Liquid fluidity index of iron ore powders.

The 1280 °C liquid-phase fluidity index varied considerably among the iron ore powder samples, as seen in Figure 3. The liquid-phase fluidity index of return C2 was the best, whereas that of low-silica A1 was only 2.95. The liquid-phase fluidity all iron ore powders used in the experiment, ordered from strong to weak, was C2 > B2 > C1 > A4 > A2 > A6 > B1 > A1 > A3 > A5.

### 3.3. Effect of Iron Ores Type on Assimilation

**Figure 4:**
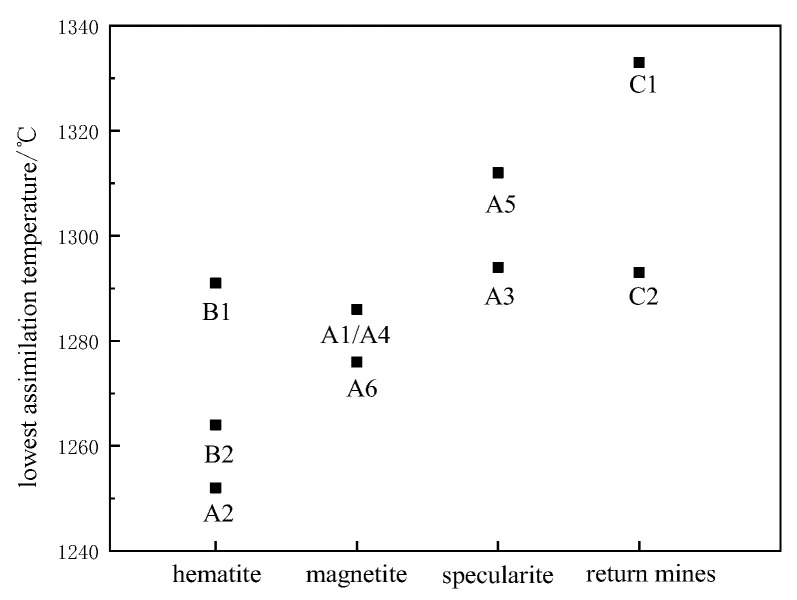
Influence of iron ore powder type on assimilation temperature.

It can be seen from Figure 4 that the minimum assimilation temperature varied according to the type of mineral powder. By comparing the types of iron ore powder and assimilation temperature, it was found that the overall assimilation order, from strong to weak, was hematite > magnetite > specularite > sinter return fines. By analyzing the structure and sintering mechanism of iron ore powder, the reasons for the influence of mineral types on assimilation temperature were determined. The assimilation temperature of hematite is low, Fe_2_O_3_ is the main iron-containing material. The thermodynamic and kinetic conditions of the reaction with CaO are good, and low-melting-point eutectics, such as calcium ferrite, are easily generated [17]. The main iron-bearing minerals in magnetite is Fe_3_O_4_, which does not react with CaO. It is necessary to oxidize Fe_3_O_4_ to Fe_2_O_3_ in an oxidizing atmosphere to produce calcium ferrite. Due to its high structural density and smooth surface, CaO is difficult to react with Fe_2_O_3_ inside the ore powder, resulting in poor dynamic conditions and a high assimilation temperature. The highest assimilation temperature of the returned ore is due to the low content of iron oxides in the returned ore, which contains Fe_3_O_4_ and FeO. Moreover, the interface between Fe_2_O_3_ and CaO is smaller, and the gangue content is high. The liquid phase is mainly a silicate phase, with reduced content of low-melting-point eutectics during sintering.

### 3.4. Effect of LOI on Assimilation

**Figure 5:**
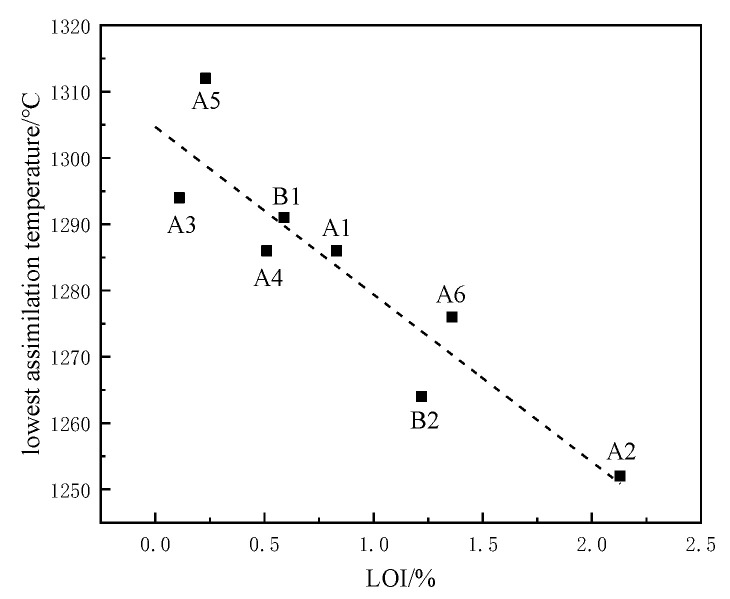
Influence of LOI on assimilation temperature.

As shown in Figure 5, the influence of LOI of iron ore powder containing crystal water on assimilation temperature was analyzed. It was found that the LOI of iron ore powder is negatively correlated with the assimilation temperature. The higher the LOI, the lower the assimilation temperature. The burning loss is mainly caused by internal structural changes caused by the decomposition of crystal water as a result of the heating of iron ore powder. Previous studies have shown that during the calcinating and heating process of iron ore powder, the crystal water in the mineral is decomposed, and a large number of cracks or even holes is generated in the internal structure. The specific surface area and porosity of each mineral increases, which can enhance the effective diffusion coefficient of CaO in iron ore powder and improve the mass transfer rate between slag system components in the binder phase of sinter [18]. At the same time, with increased contact area between the oxides, the reaction activity is increased.

### 3.5. Effect of Al_2_O_3_ Content on Assimilation

**Figure 6:**
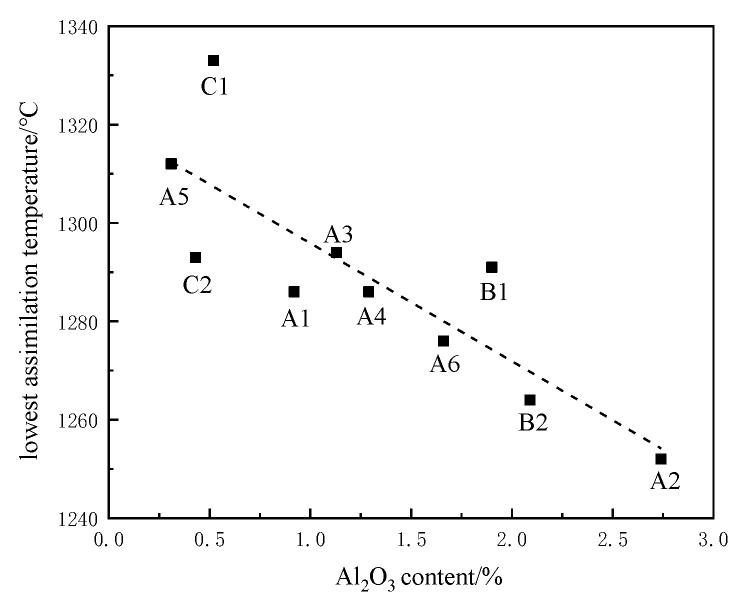
Influence of Al_2_O_3_ content on assimilation temperature.

It can be seen from Figure 6 that the assimilation temperature showed a downward trend with increased Al_2_O_3_ content. Increasing the Al_2_O_3_ content in the iron ore powder sample can reduce the assimilation temperature, which is mainly related to the liquid-phase state in the sintering process, especially in magnetite sintering. In the oxidation atmosphere of the cooling crystallization stage, magnetite is oxidized to hematite in large quantities. If the Al_2_O_3_ content in iron ore powder is increased, the surface tension of the liquid phase increases, the contact surface of oxygen increases, the transformation of hematite increases, and the formation of a composite calcium ferrite binder phase in sinter is promoted. When the iron ore powder used in the experiment was optimized, when the Al_2_O_3_ content of the ore powder was less than 0.3%, the assimilation performance of the mixed ore was improved by increasing the Al_2_O_3_ content.

### 3.6. Effect of SiO_2_ Content on Assimilation

**Figure 7:**
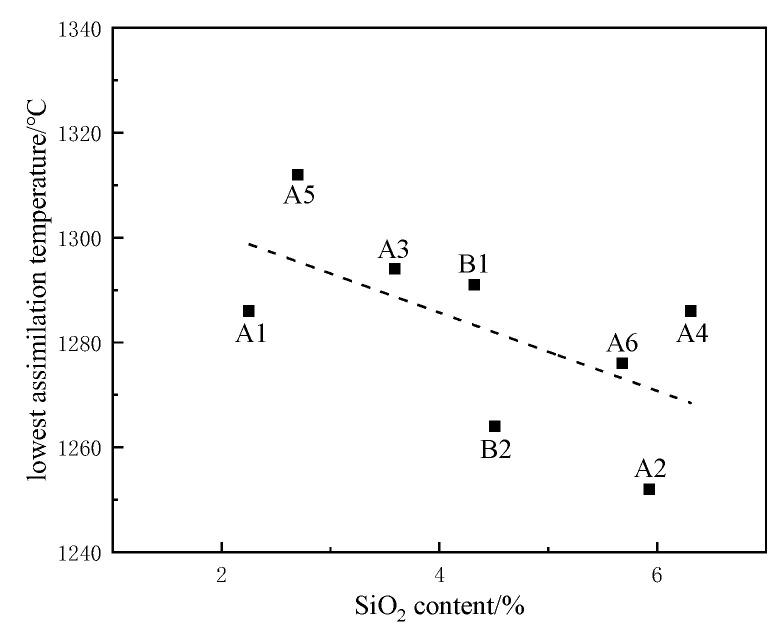
Influence of SiO_2_ content on assimilation temperature.

It can be seen from Figure 7 that the SiO_2_ content in the iron ore powder sample and the assimilation temperature showed a certain regularity. With the increase in SiO_2_ content in the iron concentrate powder sample, the assimilation temperature showed a downward trend. During sintering, SiO_2_ and Al_2_O_3_ are incorporated into binary calcium ferrite to form composite calcium ferrite (SFCA-I or SFCA). With low-silica magnetite as the main ore for high-basicity sintering, an increase in SiO_2_ and Al_2_O_3_ content promotes the formation of composite calcium ferrite (SFCA-I). In particular, SiO_2_ and Al_2_O_3_ in the form of clay have high reactivity, which reduces the minimum assimilation temperature [19,20]. The relationship between the SiO_2_ content of all iron ore powders and the assimilation temperature was analyzed as a whole. When ore blending was optimized in the experiment, when the SiO_2_ content of iron ore powder was lower than 6.0%, increasing the SiO_2_ content was conducive to reducing the assimilation temperature.

### 3.7. Effect of MgO Content on Assimilation

**Figure 8:**
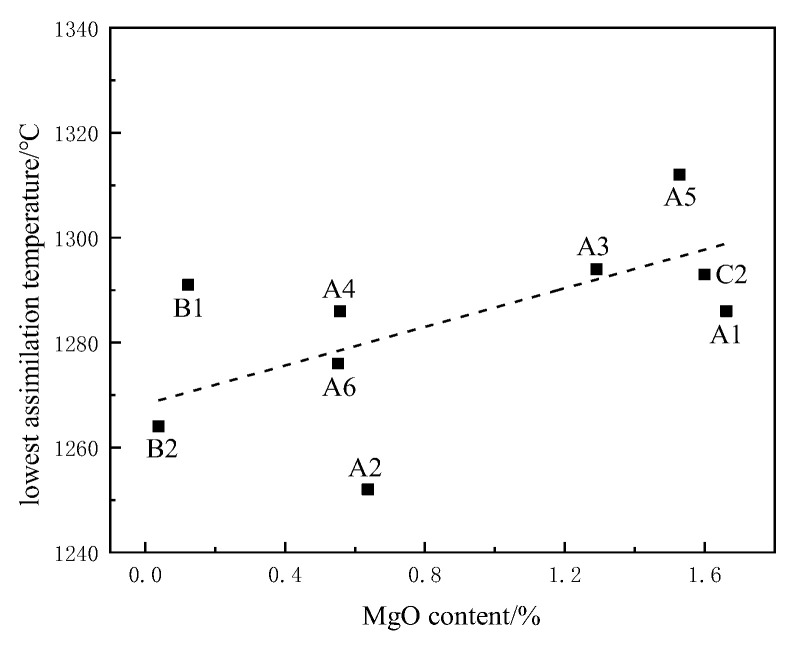
Influence of MgO content on assimilation temperature.

It can be seen from Figure 8 that the assimilation temperature in the iron ore powder sample showed a positive correlation with increased MgO content. During the sintering process, MgO forms a refractory phase with a high melting point in the solid-phase formation stage, which inhibits the formation of liquid volume of low-melting-point eutectics and leads to an increase in assimilation temperature [21]. At the same time, the addition of MgO promotes the transformation of hematite to magnetite, inhibits the formation of calcium ferrite, and reduced the bond strength and reduction of the sinter. By comprehensively analyzing the relationship between the MgO content of the iron ore powder used in the experiment and assimilation temperature, it was found that increasing MgO content in sintering materials increases assimilation temperature, and the content should be controlled below 1.6%.

### 3.8. Effect of Natural Basicity R_2_ on Assimilation

**Figure 9:**
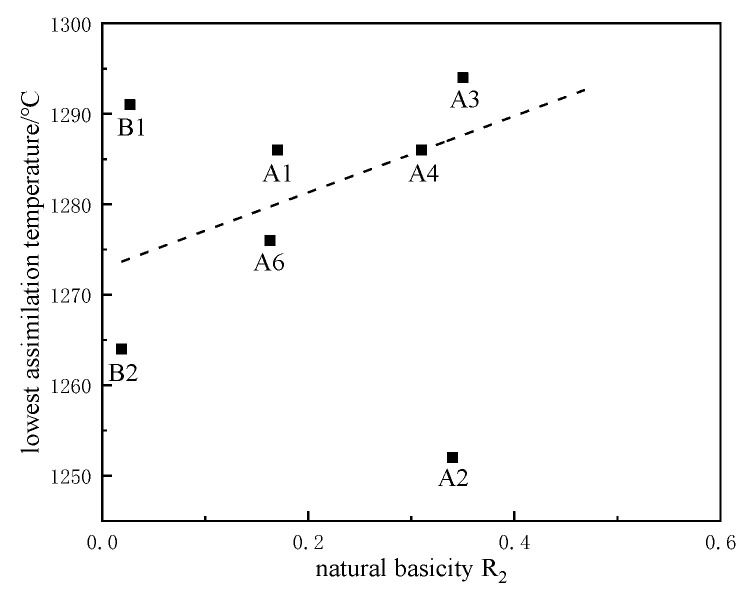
Influence of natural basicity R_2_ on assimilation temperature.

As shown in Figure 9, the natural basicity R_2_ in iron ore powder showed a certain correlation with the assimilation temperature. With the increase in natural basicity, the assimilation temperature of iron ore powder showed an upward trend. It is speculated that the iron ore powder came completely into contact with the bottom of the CaO gasket during the experiment, and the basicity on the contact surface is large. Increasing the binary basicity increases the amount of dicalcium ferrite and inhibits the formation of a liquid phase [10]. The effect of binary natural basicity on assimilation temperature was determined.

### 3.9. Analysis of Goodness of Fit of Assimilation Factors

The goodness of fit is the fitting degree of the regression line to the observation value, representing the overall fitting degree of the regression equation. Generally, the determination coefficient R^2^ is used to measure the goodness of fit. The range of R^2^ is [0, 1], and the closer the R^2^ value is to 1, the better the fitting degree of the fitted regression line to the observation value; the closer the R^2^ value is to 0, the worse the fitting degree of the fitted regression line to the observation value. Due to the difference in properties among different iron ores, not all iron ore powders used in the test were involved in the fitting process. Data points for the ten tested samples are not all included in Figure 4, Figure 5, Figure 6, Figure 7, Figure 8 and Figure 9. A few samples do not participate in the fitting process and have little effect on the overall regularity. The fitting degrees of the factors affecting the assimilation of iron ore powder are compared in Table 6.

Each variable has an obvious influence on the overall assimilation. The influencing factors of iron ore powder assimilation in this study include burning loss, SiO_2_ content, Al_2_O_3_ content, MgO content, and natural basicity R_2_. From Table 5, it can be seen that the order of significant influence on assimilation is mineral burning loss > Al_2_O_3_ content > MgO content > SiO_2_ content > R_2_. Mineral species and burning loss play a decisive role in the influence of assimilation temperature, and the influence of natural basicity on assimilation temperature is the least significant.

## 4. Optimization of Ore Blending and Sintering Quality Analysis

### 4.1. Optimizing Ore Blending Based on Assimilation

**Figure 10:**
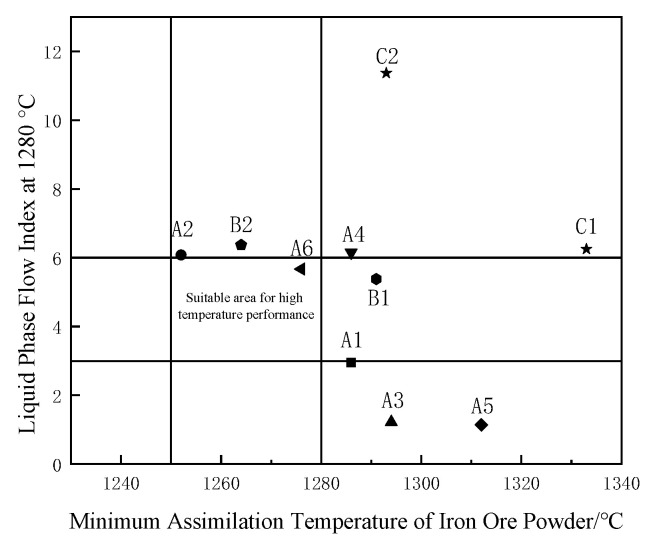
Schematic diagram of optimal collocation of iron ore powder.

The utilization of iron ore can be achieved by exploring the influencing factors of assimilation to guide ore blending, increasing the types of ore powder, and controlling the chemical composition of the mixed ore. Figure 10 shows the optimal mixture of iron ore powder used in this paper. The suitable range is marked in the figure [22]. The high-temperature characteristics resulting from the mutual blending of iron ore powder contribute to improved sinter quality [23].

It can be seen from Figure 10 that when the 10 kinds of iron ore powders in this experiment are optimized based on assimilation, A2 is complementary to C1, C2, and A5; and B2 and A6 are complementary to A1 and A3. When optimizing ore blending based on liquid-phase flow, C1 and C2 are complementary to A1, A3, and A5. Considering the assimilation and liquid-phase fluidity of iron ore powder, we selected A1 low-silicon iron ore powder as the main sintering raw material and comprehensively investigated the assimilation and liquid-phase fluidity of the resulting iron ore powder. According to the diagram of optimal matching of the iron ore powder in Figure 9, the ore-blending scheme was formulated as presented in Table 7.

### 4.2. Sintering Pot Test

**Figure 11:**
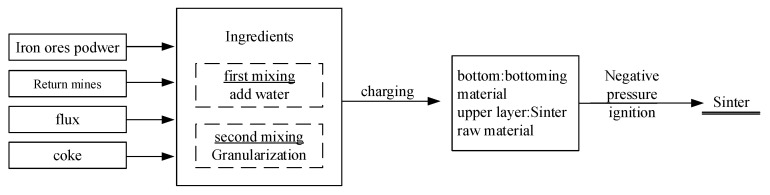
Schematic diagram of sintering pot test process.

The sintering pot test process is divided into two steps: mixing and calcinating. Mixing is further divided into two step. The first mixing step is an artificial mixing process, in which sintering raw materials, fuel, and lime are weighed and mixed in even proportions. Water is added during the mixing process for digestion. The second mixing step involves drum mixer granulation and mixing following the determination of moisture to maintain the range of 7~10%. For calcinating process, 3 kg sinter laying material was added to the bottom of the sinter pot, the sinter material was added to the sinter pot 40 kg at a time, and the ignition time was kept at 3 min. The highest temperature of the sinter material was measured as the sintering end point, and the sintering time was recorded. The sintering pot test process is shown in Figure 11.

### 4.3. Sinter Quality Index

For single-factor analysis, the following conditions were maintained to achieve the best quality of sinter: carbon content, 4.5%; water content, 8.0%; and basicity, 2.0. The quality index of the sinter is shown in Table 8 (International standard ISO 4695-1984 for reduction index and ISO 4696-1984 for low-temperature reduction degradation index).

As can be seen from Table 8, among the quality indices of sinter presented in Scheme 2, the drum index is 75.3%, the reduction index is 83.2%, and the low-temperature reduction degradation index is 81.2%. All the metallurgical performance indices are relatively good. The quality indices of sinter in Scheme 3 are lower than those in Schemes 1 and 2. With A1 iron powder as the core iron powder, an excessively fine particle size affects the granulation performance and the quality of the sinter. When A1 ore is used for sintering, there is poor air permeability, low yield after sintering, low drum strength, serious pulverization, and poor quality indicators of sinter.

By comparing the three ore-blending schemes in combination with Table 6 and Table 7, it was found that under the condition of basically equal proportions of coarse powder and miscellaneous material, the proportion of A1 and A2 iron powder has a significant impact on the quality of sinter. When the proportion of A1 iron powder increases, the sintering process deteriorates significantly, and the quality index of sinter decreases. Therefore, according to the quality indices of sinter in different ore-blending schemes, it is found that the maximum feeding amount of A1 iron ore powder is about 35%, which is more appropriate. The quality indices of sinter produced by Schemes 1 and 2 are relatively good, with a drum index higher than 75% and a low-temperature reduction degradation index higher than 80%. The quality of sinter meets the requirements of blast furnace production, indicating that Schemes 1 and 2 can guarantee the quality of sinter.

### 4.4. Microstructural Analysis of Sinter

**Figure 12:**
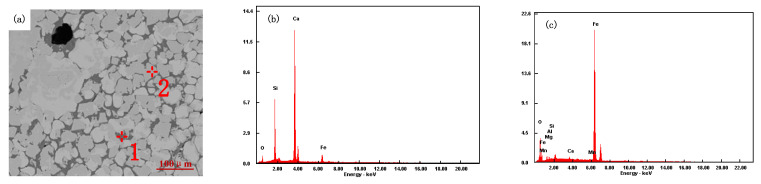
A1 SEM and EDS diagram of sinter: (**a**) A1 sintering ore, (**b**) 1, (**c**) 2.

**Figure 13:**
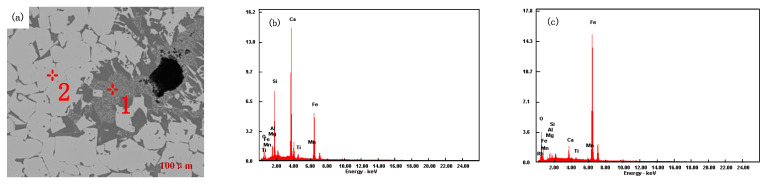
Scheme 1 SEM and EDS diagram of sinter: (**a**) Scheme 1 sinter, (**b**) 1, (**c**) 2.

**Figure 14:**
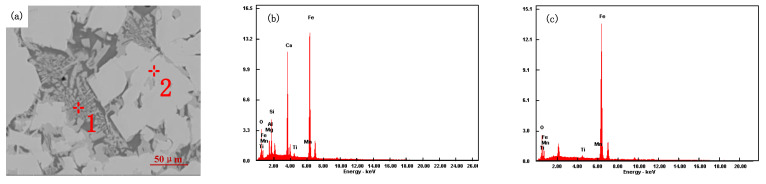
Scheme 2 SEM and EDS diagram of sinter: (**a**) Scheme 2 sinter, (**b**) 1, (**c**) 2.

**Figure 15:**
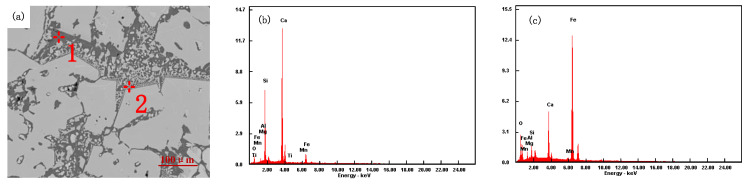
Scheme 3 SEM and EDS diagram of sinter: (**a**) Scheme 3 sinter, (**b**) 1, (**c**) 2.

Sinter samples with different ratios were analyzed by scanning electron microscopy (SEM) and energy dispersive spectroscopy (EDS), as shown in Figure 12, Figure 13, Figure 14 and Figure 15.

It can be seen from Figure 12 that heterocrystalline or semi-autocrystalline magnetite appears in the mineralogical phase (analysis point 2 in Figure 12a), with a small amount of secondary hematite after sintering of iron ore powder (A1) and a porphyritic–granular texture in mineral phases. Due to the fine particle size, low silicon content, and high assimilation temperature of A1 iron ore powder, it requires a higher temperature to generate sufficient liquid volume at high temperatures. In the sintering process, magnetite needs to be oxidized to hematite before forming a calcium ferrite liquid phase, and the formation conditions of calcium ferrite are poor. The minerals are bonded by a silicate phase (analysis point 1 in Figure 12a). The low-temperature reduction degradation index and the tumble index of the sinter are poor.

It can be seen from Figure 13 that the interlaced structure of acicular calcium ferrite and magnetite appears in the mineralogical phase after sintering of iron ore powder (Scheme 1). The microstructure of magnetite is dominated by other crystal forms (analysis point 2 in Figure 13a). A silicate slag phase intersects with calcium ferrite, and a silicate phase enwraps the needle-like calcium ferrite phase to improve the bonding phase strength (analysis point 1 in Figure 13a). The secondary hematite content is low, but the holes are large. The tumble index and the low-temperature reduction degradation index of the sinter are improved. When low-temperature reduction pulverization occurs, it can resist the stress of crystal transformation.

Figure 14 shows the mineralogical structure of the iron ore powder (Scheme 2) after sintering. In the microstructure, the mineral phase has a dendritic pilotaxitic texture. Calcium ferrite presents a dendritic or strip structure (analysis point 1 in Figure 14a), and magnetite exists in semi-autocrystal form around calcium ferrite (analysis point 2 in Figure 14a). The porosity is low, and the silicate slag phase exists in the dendritic calcium ferrite gap, which increases the binding phase strength. With the increase in hematite ratio, the content of the slag phase in the sinter increases, and the contact strength of calcium ferrite, the silicate slag phase, and magnetite increases. The sinter has good uniformity, high strength of acicular calcium ferrite, as well as a good low-temperature reduction degradation index and tumble index.

By comparing the quality indices and SEM diagrams of sinters in Scheme 1 and Scheme 2, it is found that the quality indices of both schemes are better, and there are certain differences in the bonding mechanism. The other crystalline magnetite and needle-like calcium ferrite in the Scheme 1 sinter show an interlaced corrosion structure. The strength of needle-like calcium ferrite in the bonding phase is medium, but the bonding strength of magnetite grains in the metal phase is high, which can greatly improve the strength of the sinter. The magnetite and dendritic calcium ferrite shown in the Scheme 2 sinter present an interlaced corrosion structure. The grain-bonding degree of magnetite in its metal phase is lower than that of Scheme 1 sinter, and various indices of dendritic calcium ferrite in the bonding phase are excellent, so the quality indices of Scheme 2 sinter are better.

Figure 15 shows the mineralogical structure iron ore powder (Scheme 3) after sintering. In the mineral phase structure, secondary magnetite is bonded with a silicate phase (analysis point 1 in Figure 15a), and only a small amount of calcium ferrite with network structure is distributed at the boundary of silicate and magnetite (analysis point 2 in Figure 15a). As the bonding phase is mainly a silicate phase, the low-temperature reduction degradation index and the tumble index of the sinter are poor.

## 5. Conclusions

1. The order of assimilation of the tested iron ore powders, from low to high, is: A2 > B2 > A6 > A4 = A1 > B1 > B3 > C2 > A5 > C1. SiO_2_, Al_2_O_3_, and burning loss in iron ore powder composition are positively correlated with assimilation, whereas MgO and basicity R_2_ are negatively correlated with assimilation.
2. The particle size of A1 low-silica magnetite is fine. When the appropriate proportion is not more than 35%, the hematite content increases to 17.5%, and the drum index and low-temperature reduction pulverization index of the sinter increase. Increasing the proportion of hematite with appropriate particle size can not only improve the granulation performance of sintering raw materials but also improve the liquid production and increase the content of needle-like dendritic calcium ferrite, which can help to improve the quality of the sinter. Optimizing the ore blending of low-silicon magnetite by high-temperature basic characteristics can not only improve the quality of low-silicon sinter but also improve the utilization rate of low-silicon ore.
3. The iron concentrate powder was optimized based on the assimilation performance of iron ore powder. The sintering experiment shows that the quality of sinter can be improved and the sintering process can be strengthened by ore blending, which has certain reference significance for optimizing the industrial production of low-silica sinter.

## Figures and Tables

**Table 1:** Main chemical composition of iron ore powder samples (%).

Raw Material Category	Numbering	T_Fe_	FeO	SiO_2_	CaO	MgO	Al_2_O_3_	S	P	LOI *
Domestic iron ores	A1	66.62	21.69	2.25	0.744	1.66	0.92	0.046	0.131	0.83
	A2	60.35	1.49	5.93	2.045	0.63	2.74	0.047	0.183	2.13
	A3	63.97	2.17	3.59	1.258	1.29	1.13	0.036	0.713	0.11
	A4	64.65	25.62	6.31	1.090	0.55	1.29	0.053	0.038	0.51
	A5	65.03	1.35	2.70	1.210	1.52	0.31	0.063	1.027	0.13
	A6	65.20	25.42	5.68	0.927	0.57	1.66	0.034	0.217	1.36
Foreign iron ores	B1	64.43	0.64	4.32	0.121	0.12	1.90	0.198	0.058	0.59
	B2	63.82	0.29	4.51	0.086	0.03	2.09	0.398	0.019	1.52
Sinter return fines	C1	56.16	9.45	6.34	11.360	1.43	0.52	0.209	0.051	0.33
	C2	53.45	11.37	18.26	2.489	1.60	0.43	0.141	0.270	1.27

(LOI * is loss on ignition; magnetite includes A1, A4, and A6; hematite includes A2, B1, and B2; specularite includes A3 and A5).

**Table 2:** Particle size composition of raw materials (%).

Numbering	>10 /mm	10~5 /mm	5~3 /mm	3~0.5 /mm	0.5~0.15 /mm	<0.15 /mm	Average Size /mm
A1	0.03	0.73	0.28	1.19	1.62	96.15	0.031
A2	4.75	18.95	31.65	23.30	19.26	2.09	3.26
A3	0.68	2.62	3.95	2.36	5.69	83.70	0.063
A4	3.28	18.63	10.82	35.85	28.62	2.80	2.66
A5	1.31	3.67	1.95	6.51	4.68	81.88	0.077
A6	10.29	21.37	40.69	24.32	2.92	0.41	4.34
B1	4.02	5.16	27.62	41.18	21.33	0.69	2.97
B2	2.19	18.92	38.24	25.44	11.65	3.56	3.36
C1	17.42	36.49	28.34	11.18	6.32	0.25	5.84
C2	1.08	16.22	32.46	17.39	25.57	7.28	3.93

**Table 3:** Composition analysis of coke (%).

Raw Material	Industrial Analysis (%)		
	Fixed Carbon	Ash	Volatiles
Coke	84.65	13.18	2.17

**Table 4:** Flux composition (%).

Caustic Lime	CaO	SiO_2_	MgO	LOI
Content/%	89.58	0.57	0.17	9.68

**Table 5:** Minimum assimilation temperature (°C).

Type of Iron Ore	A1	A2	A3	A4	A5	A6	B1	B2	C1	C2
Assimilation temperature/°C	1286	1252	1294	1286	1312	1276	1291	1264	1333	1293

**Table 6:** Fitting degree of each influencing factor.

Influencing Factor	LOI	SiO_2_	Al_2_O_3_	MgO	Natural Basicity R_2_
R^2^	0.83	0.36	0.69	0.62	0.13

**Table 7:** Ore-blending scheme.

Iron Ore Powder Sample	Dry Base Ratio/%		
	Scheme 1	Scheme 2	Scheme 3
A1	30.0	35.0	40.0
A2	15.0	17.5	17.5
A3	5.5	5.5	5.5
A4	5.5	5.5	5.0
A5	3.5	3.5	3.5
A6	10.0	5.0	5.0
B1	7.5	7.0	5.5
B2	10.5	8.5	5.5
C1	5.0	5.0	5.0
C2	7.5	7.5	7.5
Sun	100	100	100

**Table 8:** Quality index of sinter (%).

Index	Tumble Index/%	Yield/%	Reduction Index/%	Low-Temperature Reduction Degradation Index (+3.15)/%
Scheme 1	75.1	77.3	81.8	78.9
Scheme 2	75.3	80.1	83.2	81.2
Scheme 3	60.9	73.6	63.1	62.3
A1	46.4	56.2	50.3	49.7

## Data Availability

The data presented in this study cannot be shared at this time as the data also forms part of an ongoing study.
